# Prevalence of refractive errors in Tibetan adolescents

**DOI:** 10.1186/s12886-018-0780-8

**Published:** 2018-05-11

**Authors:** Xuehan Qian, Beihong Liu, Jing Wang, Nan Wei, Xiaoli Qi, Xue Li, Jing Li, Ying Zhang, Ning Hua, Yuxian Ning, Gang Ding, Xu Ma, Binbin Wang

**Affiliations:** 1 Department of Strabismus and Pediatric Ophthalmology, Tianjin Medical University Eye Hospital, Tianjin, China; 2 Department of Genetics, Center for Genetics, National Research Institute for Family Planning, 12 Dahuisi Road, Haidian, Beijing, 100081 China; 3 Department of Medical Genetics and Developmental Biology, School of Basic Medical Sciences, Capital Medical University, Beijing, China

**Keywords:** Refractive errors, Tibet, Abnormal rate, Myopia

## Abstract

**Background:**

The prevalence of adolescent eye disease in remote areas of the Qinghai-Tibet Plateau has rarely been reported. To understand the prevalence of common eye diseases in Tibet, we performed ocular-disease screening on students from primary and secondary schools in Tibet, and compared the prevalence to that in the Central China Plain (referred to here as the “plains area”).

**Methods:**

The refractive status of students was evaluated with a Spot™ vision screener. The test was conducted three or fewer times for both eyes of each student and results with best correction were recorded.

**Results:**

A total of 3246 students from primary and secondary schools in the Tibet Naidong district were screened, yielding a refractive error rate of 28.51%, which was significantly lower than that of the plains group (28.51% vs. 56.92%, *p* < 0.001). In both groups, the prevalence of refractive errors among females was higher than that among males.

**Conclusions:**

We found that Tibetan adolescents had a lower prevalence of refractive errors than did adolescents in the plains area, which may be related to less intensive schooling and greater exposure to sunlight.

## Background

In recent years, with the increasing educational pressure on adolescents, the incidence of eye disorders such as myopia, hyperopia and astigmatism is increasing. These common conditions can cause many inconveniences for people. Visual impairment at birth or during childhood can affect learning, communication, employment, health and quality of life, and the effects are often life-long [1]. According to a survey by Pi et al. [2], 20% of individuals with visual impairment, worldwide, are children. With the increasing prevalence of visual impairment among young people, more attention is being paid to it. In fact, it is a priority of the World Health Organization’s VISION 2020 program to control visual impairment and blindness in children [3].

Tibet is located in the southwest of China’s Qinghai-Tibet plateau. It is characterized by its high elevation (an average of > 3500 m), low air pressure and large climatic differences. There are great differences between Tibet and China’s inland cities in regional environment and eating habits. It is known that living at altitudes above 3000 m has effects on the human body [4, 5]. Accordingly, congenital heart disease, high blood pressure and other diseases in Tibet have high incidence and regional characteristics.

In the Tibetan plateau region, the incidence of eye diseases in adolescents also showed certain geographical characteristics. We found that in China, people in the plains pay more attention to the development of visual impairment than do people in Tibet and other plateau areas. Therefore, we conducted a screening study to understand the ocular health status of school-age children living on the Tibetan plateau.

## Methods

### Participants

A total of 3248 students in Tibet were enrolled in the screening. The students were from 7 primary schools and 1 secondary school in Naidong district, which averages about 3500 m above sea level. The participants in the Tibetan region are referred to as the “plateau group”.

We have included ocular screening data from 11,102 students from Tianjin Dagang as a control (“plains”) group. The average elevation of the Tianjin Dagang area is 6 m.

### Detection of ocular abnormalities

The refractive status of all students was evaluated with the Spot™ vision screener(Welch Allyn, Skaneateles Falls, NY). The screener is held approximately 1 m from the subject while they observe the display of twinkling lights and sounds. The screen reports when the subject is too far away, or too close, and shows a spinning circle and the child’s face when data acquisition is occurring. Data acquisition is usually complete within approximately 2 s. On each test, the screener measures the student’s pupil diameter, ocular alignment, binocular refraction and astigmatism axis, and stores the data. The test was conducted less than 3 times for both eyes of each student and results with best correction were recorded. The final result was determined by the instrument. The vision screener can accurately detect myopia without mydriation, so the children were not cyclopleged. Our diagnostic criteria for various eye disorders are listed in Table 1.

**Table 1:** Diagnostic criteria for various eye disorders

Age (months)	6–12	12–36	36–72	72–240	240–1200
Anisocoria	1	1	1	1	1
Anisometropia	1.5	1	1	1	1
Astigmatism	2.25	2	1.75	1.5	1.5
Hyperopia	3.5	3	2.5	2.5	1.5
Myopia	2	2	1.25	1	0.75
Vertical gaze	8	8	8	8	8
Nasal gaze	5	5	5	5	5
Radiant gaze	8	8	8	8	8
Gaze asymmetry	8	8	8	8	8

### Statistical analysis

The crude rates of myopia, hypermetropia and anisometropia were determined using direct standardization (both age and sex). Means ± standard deviation (SD), frequencies, and percentages were used to summarize the characteristics of the research subjects. The rate of each type of abnormality was compared between the two groups, using chi-square tests. The Cochran–Armitage test for trend was used to determine whether there was an association between participant age and the prevalence of disorders. All statistical analyses were performed using IBM SPSS Statistics software (version 22.0). *P*-values less than 0.05 were considered statistically significant.

## Results

We conducted eye examinations on 3248 students from primary and secondary schools in Tibet (the “plateau group”). Excluding the 19 students for whom data were missing, the plateau group had 2129 males and 1982 females. There was no significant difference in the distribution of sexes between the plateau and plains group. The mean age (± SD) in the plateau group was 12.69 (±2.88) years, which is higher than that of the plains group (see Table 2).

**Table 2:** Participant demographics

	Plateau group	Plains group
Total	3248	11,102
Gender		
Female	1558	5274
Male	1671	5740
Age (average ± SD, year)	12.69 (±2.88)	11.94 (±3.13)
Eye examination		
Normal	2322	4782
Abnormal	926	6320

The screening results were abnormal for 926 students in the plateau group (28.51% of the total). Compared with the anomaly rate of 56.93% in the plains group, the anomaly rate of the plateau group was significantly lower. Analysis of the two groups by sex showed that the anomaly rate for males in the plateau group was significantly lower than that in the plains group; the same was true for females. Finally, both groups had higher rates of anomalies among females than males.

Due to the limitations of environment and equipment, our investigation mainly focused on 6 common ophthalmic disorders: anisocoria, anisometropia, astigmatism, gaze, hyperopia and myopia. In the plateau group, there were 26 students with anisocoria (0.80%), 127 with anisometropia (3.91%), 138 with astigmatism (4.25%), 62 with gaze (1.91%), 23 with hyperopia (0.71%) and 774 with myopia (23.83%). The prevalences of anisometropia, astigmatism, gaze and myopia were significantly lower in the plateau group than in the plains group, whereas the prevalence of anisocoria in the plateau group was higher than that in the plains group. However, there was no significant difference between the two groups in the prevalence of hyperopia (Table 3).

**Table 3:** Ocular abnormalities in the two groups

Type of disorder	Plateau group (n,%)	Plains group (n,%)	Χ^2^	*p* value
Anisocoria	26(0.80)	25(0.23)	23.485	*p* < 0.001
Anisometropia	127(3.91)	1652(14.88)	278.448	*p* < 0.001
Astigmatism	138(4.25)	1290(11.62)	152.348	*p* < 0.001
Gaze	62(1.91)	791(7.12)	122.280	*p* < 0.001
Hyperopia	23(0.71)	104(0.94)	1.498	*P* = 0.221
Myopia	774(23.83)	5487(49.42)	669.251	*p* < 0.001

## Discussion

It is known that living at altitudes above 3000 m has biological effects on humans [4, 5]. The unique plateau environment comprises low air pressure, reduced oxygen, dryness, cold weather, sunshine and exposure time, intense solar infrared light, intense ultra-violet radiation, and long winters, which all have effects on the human body, in general, and on the eyes in particular [6, 7]. Because Tibet is located on the high-altitude Qinghai-Tibet plateau, the environment could be expected to affect the ocular health of people living there, but not much is known about the rates of eye diseases in the plateau region. We therefore conducted this study and used the data from the plains group to study the characteristics of common refractive errors in Tibet by comparing results between the two groups.

We found that myopia was the most prevalent abnormality in the plateau group, as well as in the plains group. In recent years, the incidence of myopia has gradually increased, attracting more attention from researchers and the public. Myopia has a serious impact on society, including on education and the economy, and it also can cause great inconvenience for patients [8]. Previous studies have shown that the prevalence of myopia in various regions of the world varies widely. In Western populations, myopia (nearsightedness) is found in one out of every three individuals [9–11]; in contrast, in selected regions of Asia, its prevalence is as high as 80% [12, 13]. High school is the high time of myopia. During this period, students can have a great deal of schoolwork to do, and previous researchers have shown that the intensity of studying closely relates to the incidence of myopia, so that could account for the high incidence of myopia in adolescents. Studies have also shown that, within a certain range, the longer the exposure to outdoor sunlight, the lower the incidence of myopia [14]. In the plateau region,, there are more hours of sunlight, and, because of the lower pressure on students, time for outdoor activities can be greater. Therefore, the students in the plateau group may be exposed to longer periods of sunshine, which could explain their lower prevalence of myopia.

Despite the high altitude of the plateau region, in the present study, the prevalence of eye disorders in Tibet was significantly lower than that in Tianjin Dagang. We hypothesize that this may be because the Tibetan children have less homework and lower scholastic pressure. The level of education in Tibet is lagging behind that in Tianjin, and students would have fewer hours in school and more time outdoors. Previous studies have shown that advising children to go outdoors at rest (for about 80 min a day) can decrease their myopia by half in 1 year [15]. Although there is no proven connection between refractive errors and educational methods or the environment, a relaxed lifestyle is likely beneficial to ocular health. In both the plateau and plains groups, the prevalence of refractive errors was higher for females than males, which may be due to girls performing fewer outdoor activities than boys do.

There are some limitations to our study. Due to logistical constraints, we could only test for common refractive errors and did not obtain information on other eye disorders or diseases. In additional, when screening for myopia, we did not have specialized inspection shadow diopter examination or clinical diagnoses. Thus we may be overestimating the prevalence of myopia, so that the rate reported here should be referred to as a “suspected myopia” rate. Finally, we did not record the children’s family relationships, so we do not know if there were cases of multiple children from the same family. Children in the same family share many lifestyle factors, which could have biased the results of the present study [2].

## Conclusion

In this high-altitude region in Tibet, the prevalence of refractive errors in adolescents was significantly lower than that in adolescents living in the plains region, which may be related to easier schooling and more exposure to sunlight.

## References

[1] Brown MM, Brown GC, Sharma S, Busbee B (2003). Quality of life associated with visual loss: a time tradeoff utility analysis comparison with medical health states. Ophthalmology.

[2] Pi LH, Chen L, Liu Q, Ke N, Fang J, Zhang S, Xiao J, Ye WJ, Xiong Y, Shi H (2012). Prevalence of eye diseases and causes of visual impairment in school-aged children in western China. Journal of epidemiology.

[3] Gilbert C, Foster A (2001). Childhood blindness in the context of VISION 2020--the right to sight. Bull World Health Organ.

[4] Wang GQ, Bai ZX, Shi J, Luo S, Chang HF, Sai XY (2013). Prevalence and risk factors for eye diseases, blindness, and low vision in Lhasa, Tibet. International journal of ophthalmology.

[5] Gallagher RP, Lee TK (2006). Adverse effects of ultraviolet radiation: a brief review. Prog Biophys Mol Biol.

[6] Guo B, Lu P, Chen X, Zhang W, Chen R (2010). Prevalence of dry eye disease in Mongolians at high altitude in China: the Henan eye study. Ophthalmic Epidemiol.

[7] Bali J, Chaudhary KP, Thakur R (2005). High altitude and the eye: a case controlled study in clinical ocular anthropometry of changes in the eye. High altitude medicine & biology.

[8] Saw SM, Katz J, Schein OD, Chew SJ, Chan TK (1996). Epidemiology of myopia. Epidemiol Rev.

[9] Kempen JH, Mitchell P, Lee KE, Tielsch JM, Broman AT, Taylor HR, Ikram MK, Congdon NG, O'Colmain BJ (2004). Eye diseases prevalence research G: the prevalence of refractive errors among adults in the United States, Western Europe, and Australia. Arch Ophthalmol.

[10] Lee KE, Klein BE, Klein R, Wong TY (2002). Changes in refraction over 10 years in an adult population: the beaver dam eye study. Invest Ophthalmol Vis Sci.

[11] Chang PY, Yang CM, Yang CH, Huang JS, Ho TC, Lin CP, Chen MS, Chen LJ, Wang JY (2005). Clinical characteristics and surgical outcomes of pediatric rhegmatogenous retinal detachment in Taiwan. Am J Ophthalmol.

[12] Shimizu N, Nomura H, Ando F, Niino N, Miyake Y, Shimokata H (2003). Refractive errors and factors associated with myopia in an adult Japanese population. Jpn J Ophthalmol.

[13] Wong TY, Foster PJ, Hee J, Ng TP, Tielsch JM, Chew SJ, Johnson GJ, Seah SK (2000). Prevalence and risk factors for refractive errors in adult Chinese in Singapore. Invest Ophthalmol Vis Sci.

[14] Wang Y, Ding H, Stell WK, Liu L, Li S, Liu H, Zhong X (2015). Exposure to sunlight reduces the risk of myopia in rhesus monkeys. PLoS One.

[15] Wu PC, Tsai CL, Wu HL, Yang YH, Kuo HK (2013). Outdoor activity during class recess reduces myopia onset and progression in school children. Ophthalmology.

